# A Ubiquitous Sensor Network Platform for Integrating Smart Devices into the Semantic Sensor Web

**DOI:** 10.3390/s140610725

**Published:** 2014-06-18

**Authors:** David Díaz Pardo de Vera, Álvaro Sigüenza Izquierdo, Jesús Bernat Vercher, Luis Alfonso Hernández Gómez

**Affiliations:** 1 Universidad Politécnica de Madrid, GAPS-SSR, E.T.S.I. Telecomunicación, Avda. Complutense 30, 28040 Madrid, Spain; E-Mails: ajsigu@gmail.com (A.S.I.); luisalfonso.hernandez@upm.es (L.A.H.G.); 2 Telefónica I+D, Ronda de la Comunicación, s/n, 28050 Madrid, Spain; E-Mail: bernat@tid.es

**Keywords:** Sensor Web, Semantic Sensor Web, smart devices, Telco Platform, Ubiquitous Sensor Networks, next-generation networks

## Abstract

Ongoing Sensor Web developments make a growing amount of heterogeneous sensor data available to smart devices. This is generating an increasing demand for homogeneous mechanisms to access, publish and share real-world information. This paper discusses, first, an architectural solution based on Next Generation Networks: a pilot Telco Ubiquitous Sensor Network (USN) Platform that embeds several OGC^®^ Sensor Web services. This platform has already been deployed in large scale projects. Second, the USN-Platform is extended to explore a first approach to Semantic Sensor Web principles and technologies, so that smart devices can access Sensor Web data, allowing them also to share richer (semantically interpreted) information. An experimental scenario is presented: a smart car that consumes and produces real-world information which is integrated into the Semantic Sensor Web through a Telco USN-Platform. Performance tests revealed that observation publishing times with our experimental system were well within limits compatible with the adequate operation of smart safety assistance systems in vehicles. On the other hand, response times for complex queries on large repositories may be inappropriate for rapid reaction needs.

## 1. Introduction

Sensors of the physical world, computing systems and information networks are the basic elements of a rapidly approaching future in which the realms of physical objects and digital information will merge, paving the way to a new generation of smart applications in a wide range of areas such as eHealth, logistics, intelligent transportation, environmental monitoring, smart grids, smart metering and home automation. The Internet of Things (IoT) [1] and related ideas picture smart devices and connected objects acquiring meaningful information about their environment and communicating it to other devices, objects and people, through a “global network infrastructure based on standard and interoperable communication protocols where physical and virtual things are seamlessly integrated into the information network” [1]. For this vision to come to fruition advancements are needed in two fundamental areas: first, the integration and accessibility of the heterogeneous and ever growing number of sensor and actuator networks; and second, the meaningful organization of the ocean of data coming from these networks and the derivation of further knowledge from it.

The Sensor Web [2] is one of the more promising concepts for the development of the IoT. The term Sensor Web refers to an infrastructure with automatic mechanisms to allow applications to discover and access particular information provided by sensors networks over the Internet. But the success of the IoT depends strongly on the existence of global standards to avoid the inefficiency of developing particular solutions for specific applications. The Open Geospatial Consortium (OGC) has developed a set of Sensor Web Enablement (SWE) standards providing common interfaces and metadata encodings to discover and access data provided by heterogeneous sensor networks and smart devices [3]. However, as stated by the recently created Standard Working Group on Sensor Web for IoT (www.opengeospatial.org/projects/groups/sweiotswg) (SWE_IoT_SWG), these standardization efforts still need to be extended to all the interfaces that can be identified between the lower layer, represented by sensor networks and smart devices, and the upper layer of final applications. The SWE_IoT_SWG identifies interfaces between three different layers: a Sensor Layer (*i.e.*, sensor networks and smart devices), a Sensor Web Layer and an Application Layer (*i.e.*, final IoT applications). In our view, the OGC SWE represents a mature standard interface between the Sensor Web Layer and the Application Layer. Universal standards for the interface between the Sensor Web and the underlying Sensor Layer, however, are still an open issue [4–7].

International telecommunication standardization organizations, including the ITU, ETSI and 3GPP [8–10], are involved in a number of IoT initiatives working towards standard interfaces between the Sensor Layer and the Sensor Web. Seeking to contribute to these initiatives, in this paper we present a conceptual platform within a global Telco framework, which relies on Next Generation Networks to interconnect the Sensor Layer and the Sensor Web, and on OGC SWE standards to provide interfaces between Sensor Web and IoT applications. More specifically our Telco IoT Platform was designed following an idea that the ITU has identified as important in the standardization process to create the IoT: the Ubiquitous Sensor Network (USN). The ITU defines a USN as “a conceptual network built over existing physical networks which makes use of sensed data and provides knowledge services to anyone, anywhere and at anytime, and where the information is generated by using context awareness” [11]. To better understand the key elements in the Telco IoT Platform we propose, it is useful to review the main components of a USN:

- USN Applications and Services platform, which enables the effective use of a USN in a given application or service.
- USN Middleware, including functionalities for sensor network management and connectivity, event processing, sensor data mining, *etc.*
- Network infrastructure, mainly based on Next Generation Networks (NGN).
- USN Gateway: a node which interconnects sensor networks with other networks.
- Sensor networks.

**Figure 1.**
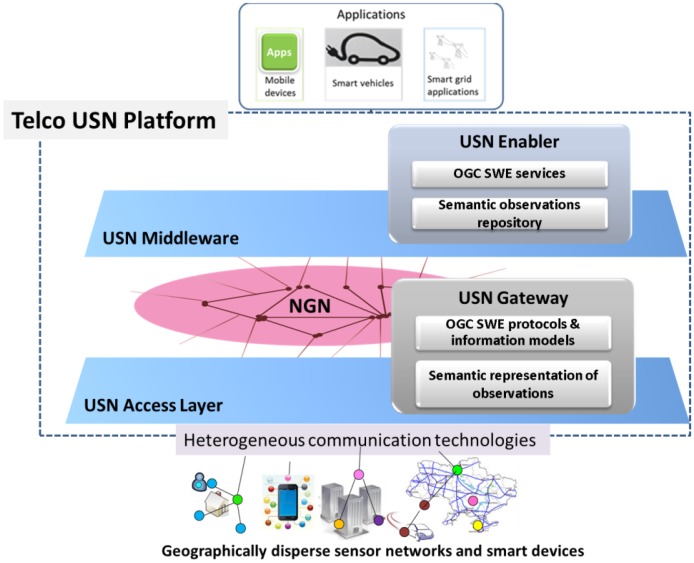
Conceptual architecture of a Ubiquitous Sensor Network Platform to connect applications (e.g., smart devices) with sensor and actuator networks. (Components of the proposed semantic extension are also shown).

It is also important to underline that a USN is not a physical network, it is a conceptual architecture that makes use of existing networks. As Figure 1 illustrates, the USN has at its core a Next Generation Network (NGN) infrastructure, which provides ubiquitous connectivity and all the control and management network capabilities. This is the communications infrastructure, on top of which the Sensor Web layer can be fully integrated as a part of the USN Middleware.

Based on this idea, the USN-based platform (USN-Platform) we propose integrates OGC SWE functionalities into the USN middleware through what we refer to as a USN-Enabler (see Figure 1). Furthermore, with the USN-Platform built around NGNs, the gap between the Sensor Layer and the Sensor Web Layer is bridged by means of USN-Gateways. As shown in Figure 1, it is through USN-Gateways that heterogeneous and geographically dispersed sensor networks and smart devices can be interconnected, discovered, accessed and controlled, using protocols and information models standardized by OGC SWE.

Once the USN-Platform is in place to bridge the underlying heterogeneity of communication technologies, the opportunity arises for smart applications to combine and reason with different kinds of information from diverse sources. This is the second challenge we identified in the beginning.

Semantic Web [12] technologies, an evolving extension of the current Web, are intended to provide well-defined and machine accessible information that is shareable across applications. Combining the Sensor Web and the Semantic Web we arrive at the notion of the Semantic Sensor Web (SSW). These technologies are being developed to provide formal descriptions of the entities related to sensor data, in particular the observations of real world features and the sensors that can capture them. For this purpose the W3C has produced a Semantic Sensor Network Ontology (SSNO) [13], which is a broad and complex conceptual structure.

While acknowledging the central role that the Semantic Sensor Web is set to play in the future IoT, in this paper we present work that explores a first step of drawing the USN-Platform toward SSW technologies. It is, to our knowledge, the first time semantic technologies are included in a Telco IoT Platform. Nevertheless, we acknowledge that the Platform extension we have considered only handles semantic descriptions of observations (*i.e.*, the data given by sensors) as a relatively simple set of concepts and relations following the SemSOS approach [14]. We will refer to the encoding of sensor observations in terms of said concepts and relations as *semantic annotation*. To accommodate this semantic representation of observations, the USN-Platform was extended with two elements (see Figure 1): one to enable the communications layer (USN-Gateway) to handle the semantically annotated sensor observations, and another in the service layer (USN-Enabler) to store these observations and allow applications to reason with the accumulated data. With this semantic extension the applications may themselves publish processed information that results from reasoning with sensor data and also with observations provided by human users through appropriate interfaces.

With a “semantically enhanced” USN-Platform we aim to tap into the great potential that the exchange of semantic information can offer for the development of future applications for the increasing number of smart devices in circulation (mobile phones, connected vehicles, home appliances, *etc.*). In particular, we sustain the view that smart devices can become not only consumers of sensor or real-world data but also, through interaction with their environment, relevant producers of enriched information for the Semantic Sensor Web. In this paper we illustrate the different technologies constituting the USN-Platform with an electric car scenario in which a smart car acts both as consumer and producer of the information on the Semantic Sensor Web. This smart-car application scenario has been implemented experimentally.

The rest of the paper is organized as follows: Section 2 presents the architecture we propose to implement the conceptual model to connect heterogeneous applications and sensor networks: the Telco Ubiquitous Sensor Network (USN) Platform. In Section 3 we describe an extension of this Platform employing basic Semantic Web technologies to provide more powerful mechanisms (accessing, annotating, publishing and sharing) to integrate smart devices into the future Semantic Sensor Web. In Section 4 we present an example scenario of a smart electric car exchanging information with a smart grid, featuring sensor and human-generated information that can be semantically annotated and combined to make inferences. An experimental implementation of this scenario is described in Section 5. Section 6 discusses the results of semantic processing efficiency evaluation tests performed on the experimental system. Conclusions and future challenges are presented in Section 7.

## 2. The Ubiquitous Sensor Network Platform

The vision of providing sensor data for services is not new and there are many initiatives working in that direction. Examples of projects in the environmental monitoring area are the SANY (www.opengeospatial.org/ogc/regions/SANY) (based on OGC standards) and ENVIROFI (www.envirofi.eu/FrequentlyAskedQuestions.aspx) projects. OGC is also working in the direction of extending the current SWE standards towards the IoT (www.opengeospatial.org/projects/groups/ sweiotswg). In addition to the OGC SWE framework considered for the USN-Platform presented here, we note that there are a number of other Sensor Web initiatives that can be described as centralized web portals, such as Axeda (www.axeda.com), Xively (xively.com/whats_xively/) and Sensorpedia (www.sensorpedia.com). These Sensor Web portals offer APIs for registering and discovering sensors, uploading sensor data, as well as querying inserted data. Other noteworthy open architectures with the goal to make physical world information available for smart applications have and are being developed in different international projects such as SENSEI (www.sensei-project.eu/), IoT-A (www.iot-a.eu/ public/front-page), Artemis SOFIA (www.sofia-project.eu/), Harvard HOURGLASS (www.eecs.harvard.edu/∼syrah/hourglass/index.shtml), Global Sensor Networks (GSN) (sourceforge.net/ apps/trac/gsn/), OpenIoT (www.openiot.eu), or SOCRADES (www.socrades.eu/Home/default.html).

The Ubiquitous Sensor Network (USN) Platform, which was first proposed in [15], is organized conceptually in two layers: a communication layer and a service layer. In the architecture presented here there are two main logical components that correspond to each of these layers, respectively: USN-Gateways, which facilitate the integration of heterogeneous IoT information providers through the Next Generation Networks; and a USN-Enabler, which implements the sensor service layer, based primarily on OGC-SWE specifications. We describe these two components after a brief introduction to NGNs, their function in the USN-Platform, and the benefits for developing future IoT architectures that can be derived from their use.

### 2.1. Technological Context and NGNs

The increasing diversity of communication technologies, either IP-based (such as 6LoWPAN or CoAP) or non ip-based technologies which are often proprietary (e.g., ZigBee or KNX), has motivated the research of new solutions to bridge the gap between Sensor Networks and the Sensor Web. For instance, the SensorBus [4] has been proposed (as a part of the open source implementation of OGC SWE by the North 52° (52north.org/) community) as an intermediary layer between geosensor networks and the Sensor Web. A particular SensorBus implementation based on Twitter has been proposed in [5] that allows reusing useful Twitter functionalities such as authentication and scalability management. Another extension of SensorBus, called SensorAdapter, has been presented in [6] as a real-time communication adapter designed to allow simultaneous communication between different sensors protocols and the data service layer in the Sensor Web. Also recently [7], a web service proxy has been proposed to link the low level sensor networks to the high level SWE. In a similar direction, relevant European projects have addressed this challenge of bridging the gap between sensor networks and the Sensor Web, for example, the SANY project [16] has proposed the SensorSA Data Acquisition System (SensorSA DAS) designed as a network capable appliance which allows seamless integration of various sensors using communications based on OGC SWE interfaces.

Current services and technologies to access real-world information are typically vertical, application-specific solutions. However, cross-domain IoT platforms foster the increasingly efficient creation of new services by enabling the re-use of deployed infrastructures, in a competitive scenario where multiple applications can choose dynamically among different infrastructures based on their availability, security, quality of service, *etc.*

The main contribution of our USN-Platform is to integrate Sensor Networks and Sensor Web around the Next Generation Network (NGN) [17], which is a packet-based network capable of making use of multiple broadband, QoS-enabled transport technologies, and in which service-related functions are independent from underlying transport-related technologies. NGNs embody a good set of principles that can help the development of IoT applications at two levels: the integration of heterogeneous IoT devices and the facilities offered to develop services. Sensor and Actuator Networks (SANs) will be used in a broad set of heterogeneous application scenarios leading to a big variety of deployments with completely different communication infrastructures, protocols, speeds, latencies, *etc.* For example, in some cases SANs could be available through a broadband Internet connection, while in others they could be accessible through GRPS connectivity. One of the most crucial aspects of NGNs is the homogeneous support of multiple mobile and fixed access networks such as hybrid fiber-coaxial (HFC), power line communications (PLC), satellite, GPRS, CDMA, GSM, HSDPA or xDSL. This characteristic makes it key for supporting highly diverse deployment scenarios, e.g., a Sensor Network connected to an xDSL line or a sensor attached to a mobile phone. Moreover, since these networks are spread almost everywhere, they offer a huge range of geographic coverage for potential deployments. But even more importantly, NGNs provide a set of core functions needed for developing final customer services like identification, authorization, authentication, privacy, security or quality of service (QoS), accounting, billing, service discovery and mobility. These functions are critical for the deployment of IoT services.

This notion of interconnecting sensor networks over Telco infrastructures has been proposed before. The EU project e-SENSE [18] can be identified as one of the first references proposing the integration of sensor networks into IP based telecommunication networks, but does not account for Sensor Web infrastructures. Other initiatives have provided insights regarding the problems of integrating wireless sensor networks into telecommunication platforms, e.g. [19], but again without considering the complementary progress in new Sensor Web activities.

### 2.2. The USN-Gateway

**Figure 2.**
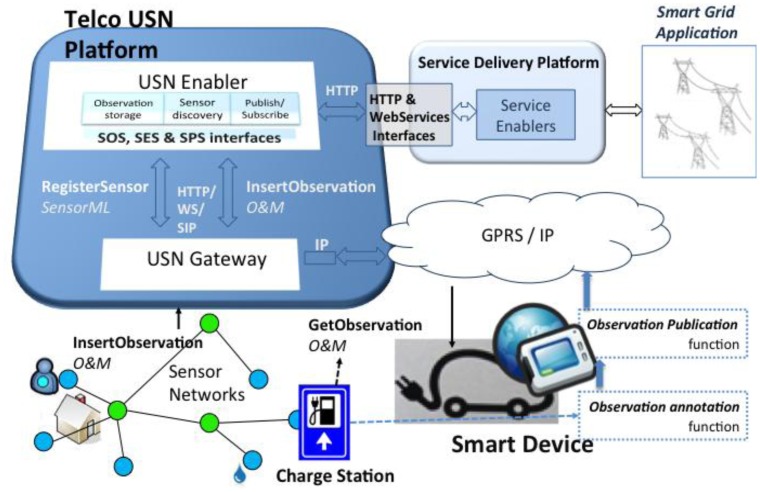
Integration of Smart Devices into a Telco USN-Platform.

Figure 2 shows how the USN-Platform interconnects smart applications and sensor networks. USN-Gateways act as the interconnection points between IoT infrastructures and the Enabler. They provide two main functions: communication protocol adaptation (from the particular sensor protocols, such as MQTT, to HTTP, WebServices and/or SIP) and sensor data adaption (from the particular protocols to OGC SWE information models—SensorML and O&M (see Section 2.3 below)). By virtue of these two sets of conversions performed by the USN-Gateways, the USN-Enabler can be independent of the particular networking and data technologies used in the sensor networks.

In this view, smart devices, which may be deployed in heterogeneous environments, connect directly or through gateways to a core NGN using a variety of access technologies (GPRS, UMTS, WiFi, *etc.*). In many cases the devices can use their own communication capabilities to establish an IP connection with external USN-Gateways, or they can even have the USN-Gateway itself integrated in them. In our implementation a major scalability-enabling point is that once smart devices are connected to USN-Gateways, they are managed just like any other user equipment in the IMS (IP Multimedia Subsystem). The IMS is the control component of the NGN.

In our USN-Platform smart devices can be managed in similar fashion to how Telco platforms regularly handle millions of user terminals today. In addition, they automatically benefit from the range of interesting features that NGNs offer, which were mentioned previously. Particularly notable and important are those related to security and authentication (functions not offered by the OGC standards). NGNs also offer provisioning, multi-network support, and extended and flexible session control. This provides an excellent basis for features such as load balancing, session continuity, and quality adaptation. All of these functions, together with a list of well-known enablers (for presence, location, instant messaging, *etc.*) [20], make it possible to deliver high quality service and to provide a deployment platform in which final services can be offered in a broad variety of secure and reliable environments (operator centers, client location, smart devices, *etc.*).

The internal design of the USN-Platform has further interesting features that increase the advantages of this architectural proposal. For example, the platform is a transactional system; it is reliable against application disconnections (applies retrial policies for notifications); it supports store & forward of configuration commands; it is scalable, *etc.* Furthermore, the platform has been engineered in such a way that it is capable of running not only on an NGN but also in other environments, and it can be deployed over broad IP infrastructures. However, in these other environments the additional features provided by the NGN (mainly security-related) would have to be provided by other (external) components.

### 2.3. The USN-Enabler

Integrated in the middleware element of the USN is the USN-Enabler. The mission of the USN-Enabler, the main component of the USN-Platform, is to manage the infrastructures and assist IoT applications by providing a set of OGC-SWE services which facilitate the integration of smart devices with the Sensor Web. This allows the implementation of sensor discovery, access and data publication services with enhanced properties.

**Figure 3.**
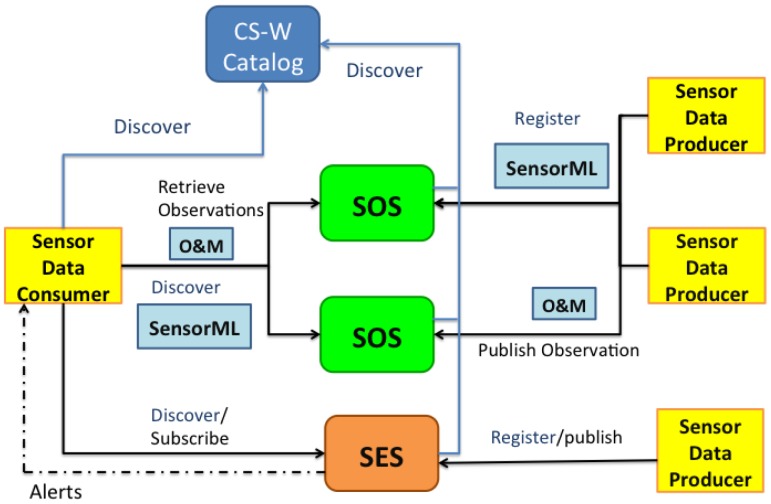
Sensor Web Infrastructure example with services defined by the OGC-SWE.

Figure 3 shows an example of Sensor Web Infrastructure using some of the services proposed by the OGC-SWE. In the OGC model applications do not interact directly with the data producers. Instead they rely on OGC services (two are shown in the example in Figure 3: the Sensor Observation Service (SOS) and the Sensor Event Service (SES)). Data producers must first register to these services in order to be discoverable by smart devices and applications. Since multiple instances of these services can co-exist, applications must first look up which of them is the most suitable provider of the desired information in its current context of use. This sensor information discovery is done through OGC Catalogues [21], as shown in Figure 3 (the CS-W: Catalogue Service). The OGC Catalogue is an optional component in the SWE architecture, but it is the only standard mechanism that allows clients to discover adequate service providers for the data they are interested in. Once the appropriate source is found, applications can access specific sensor information by querying the service.

Two interaction mechanisms are available to access sensor information: a pull-based system, in which applications query the information when needed; and a push-based mode, in which applications subscribe to alerts to be notified when certain conditions or situations occur. For the first type of interaction the OGC-SWE defines the Sensor Observation Service (SOS) [22], while for the second it offers a Sensor Event Service (SES) [23]. The SES interface follows a publish/subscribe model where a sensor can send (advertise) a current observation value as an alert (for example, the number of vehicles per hour at a road intersection). A consumer application can subscribe to receive custom alerts when the sensed data meets set criteria (e.g., a measurement above a threshold, or occurring in a location area or a time interval).

SWE specifications rely on two main information models to describe the sensor systems and their related observations or sensed measurements: SensorML and Observations & Measurements (O&M), respectively. SensorML allows the definition of different types of sensors and sensor systems: simple sensors nodes, processors (components that process data to derive new information), complex sensor systems (e.g., earth observation satellites), *etc.* The O&M language defines a domain-independent conceptual model for the representation of sensed spatiotemporal data. Both standards offer syntactic uniformity for sensor and data representation respectively. However, the same item (e.g., a sensor) can be represented in different ways using these standards, which poses a challenge to achieve interoperability. Beyond the functionality specified by the OGC-SWE, the underlying NGN architecture makes it possible to enhance these services, as provided by the USN-Platform, as follows:

- Simpler interactions for information providers: If an information provider wishes to offer data through more than one OGC service (e.g., SOS and SES, for storage and event management functions) it needs to register and publish the same information several times, using different protocols. This is inefficient and unreliable (race conditions and information inconsistencies may occur, among other undesirable effects). In the USN-Platform information is published and registered only once, using the SOS functions, and then made available to the other OGC services.
- An improved discovery mechanism: Standard OGC discovery for all services is defined through a PULL-based discovery mechanism that retrieves a list of results specifying some aspects of the sensors but not a full description, nor is a filter criterion allowed. In the USN-Platform the PULL-based interaction allows filtering based on the content of the fields in SensorML. In addition to query results, the USN-Enabler can return SensorML descriptions and enable PUSH-based interaction by allowing applications to subscribe to alerts of changes in SensorML documents that match certain criteria.
- Improved subscription: SES does not allow subscriptions to changes in SensorML descriptions; the USN-Platform does. The platform is also able to increase the granularity of notification channels to the level of gateways (e.g., all sensors accessible through a particular gateway) or even down to a single sensor (e.g., all measurements provided by sensor “X”).
- Refined observation retrieval: SOS defines a PULL-based mechanism to recover information regarding observations that meet certain criteria (e.g., recover only observations collected from a specific sensor, related to a particular feature of interest, and obtained under given spatial or temporal restrictions). However, more complex conditions (such as temp >25 °C) are not specifiable. An extension has been defined in the USN-Platform that allows using the same filtering criteria as for PUSH-based interactions.

### 2.4. How the USN-Platform Works for Smart Devices

Once connected to USN-Gateways, smart devices, together with any attached local sensors, are advertised in the USN-Enabler just like any other sensor system. More specifically, as depicted in Figure 2, the USN-Gateway establishes an HTTP connection to add a new basic SensorML description of the smart device with the *RegisterSensor* operation defined using the SOS interface. In our electric car example, which will be described in Section 4, the sensor system is a particular electric car that provides battery level readings and precise charging information (mainly the time and the charging station chosen).

In order for a smart device to become a Sensor Web producer it must implement two additional functions: observation *annotation* and observation *publication* (again, see Figure 2). The observation annotation function receives data from the smart application and generates O&M annotations, adding spatial and temporal information and specifying the type (e.g., temperature, time), the generator (ID of the sensor) and the entity of interest (e.g., the electric car). Then the observation publication function is used to publish it in the Observation Storage of the USN-Platform. This function uses the *InsertObservation* operation provided by the USN-Enabler.

Through these functionalities that the USN-Enabler provides, smart devices and other applications can look for and consume real-world information available in the Sensor Web.

### 2.5. Field Tests

Built on NGN infrastructures, the USN-Platform offers scalable message routing mechanisms across several administrative domains, to support smart device communications in similar fashion to how it is done for the millions of user terminals any Telco Platform is designed to deal with. The use of NGNs supporting context-aware applications in connected car scenarios has already shown improved performance in routing protocols compared to traditional vehicular communications over Vehicular Ad-hoc Networks (VANETs) [24]. According to Lequerica *et al.*, the use of NGNs to provide backup for data communication, as well as efficient mechanisms for the dissemination of relevant information, can provide a 20% increase in packet delivery ratio compared to VANET protocols, under situations of constrained mobility (e.g., streets without enough traffic density for uninterrupted V2V communication).

We have already evaluated our Telco USN-Platform (without the semantic extension, which will be discussed in the next section) in several field trials for smart metering, smart places, and environmental monitoring [25]. Particularly noteworthy is its current deployment under the SmartSantander (www.smartsantander.eu/) EU Project: an IoT testbed facility that is being developed in a Smart City environment aiming to integrate up to 20,000 IoT devices by the end of 2013. So far it has integrated around 2500 sensors of different types providing mainly environmental (temperature, relative humidity, CO, noise level, *etc.*) and parking information (sensing whether each particular parking space is free or taken). The USN-Platform implementation used in the development of services for SmartSantander is based on a scalable distributed architecture currently composed of a reduced set of specialized servers, and is suitable to provide horizontal support for multi-service deployments. This set-up is capable of managing information from more than 20,000 sensors providing observations every minute.

## 3. Extending the Telco USN-Platform towards the Semantic Sensor Web

The World Wide Web Consortium's (W3C) Semantic Sensor Network Incubator Group (SSN-XG) (http://www.w3.org/2005/Incubator/ssn/) has observed that OGC-SWE specifications provide “limited interoperability and data exchange based on XML and standardized tags. However, they do not provide semantic interoperability and do not provide a basis for reasoning that can ease development of advanced applications” [13]. To overcome this limitation, and in the same vein as the approach proposed by the W3C's SSN-XG, we integrated several W3C semantic technologies into our USN-Platform.

The SSN-XG has focused its activities on both the development of ontologies to describe sensors (for example, mapping OGC standards to OWL) and the extension of XML-based sensor and observation markup languages to support semantic annotations (facilitating the integration with linked open data applications relying on semantic web technologies like RDF and SPARQL). To progress towards these Semantic Sensor Web benefits, the base USN-Platform was extended to include two major developments:

(1) New capabilities to manage semantically annotated observations coming from smart devices (mobile phones, connected vehicles, home appliances, interactive urban infrastructures, *etc.*).
(2) Extension of the Observation Storage in the USN-Enabler so that applications can make use of functionalities offered by Semantic Web repositories: semantic queries, intelligent search, inference, reasoning, *etc.*

### 3.1. Semantic Annotation of Smart Device Observations

**Figure 4.**
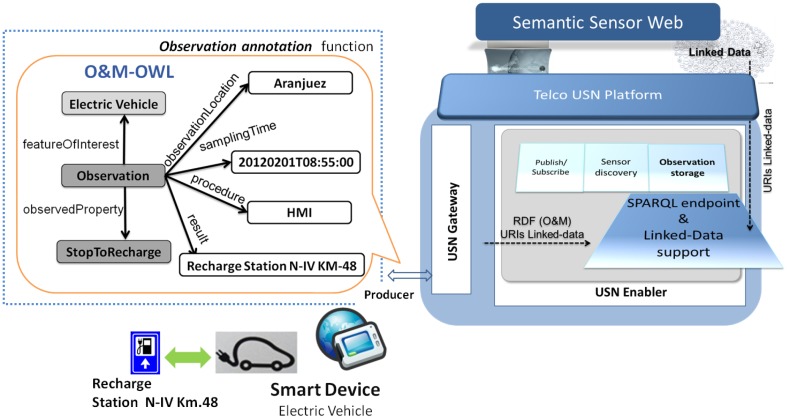
Adding semantic features to the Smart Device and Telco USN-Platform.

**Figure 5.**
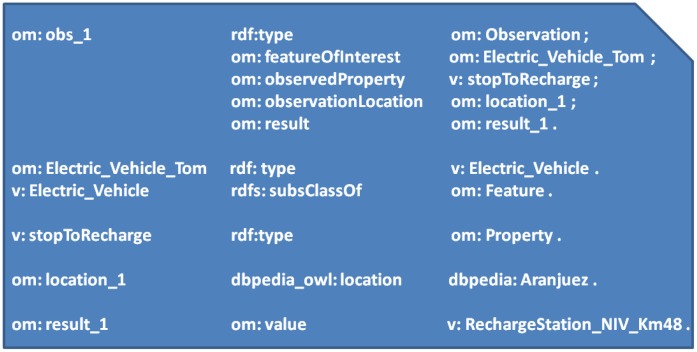
Example of an Electric Vehicle observation using O&M-OWL.

To extend the USN-Platform some way towards the Semantic Sensor Web paradigm we first chose an annotation scheme for the observations generated by smart devices. Developing OWL customized ontologies for OGC Observations can be very complex [26]. In the work presented here, however, we have limited the task to implementing a simple semantic representation of the OGC O&M language. The representation in question consists in the encoding of O&M in OWL, henceforth referred to as O&M-OWL. (The interested reader can find more details on the mapping from O&M to O&M-OWL in [14].) Figure 4 shows the main O&M-OWL concepts and relations, namely, *featureOfInterest*, *observedProperty*, *procedure*, *observationLocation*, *result* and *samplingTime*. Figure 5 shows an example of a semantically annotated observation expressed as a set of RDF triples, corresponding to the electric-car scenario that will be described in Section 4.

After choosing an annotation scheme for smart device observations, two functional areas were extended in the USN-Platform (see Figure 4):

(1) USN-Gateway functions, to manage semantically annotated observations in two ways: either by supporting already annotated measurements or by automatically annotating the observations generated by smart devices.
(2) The *InsertObservation* operation provided by the USN-Enabler, to receive these semantically annotated observations, which are then stored in a Semantic Web repository, as we describe below.

### 3.2. Extending the Observation Storage with a Semantic Web Repository

The Observation Storage entity of the USN-Enabler was extended to make RDF observations received by the USN-Platform from smart devices (such as the human-generated observations received from HMI systems) shareable with third party applications. As represented in Figure 4, a SPARQL (http://www.w3.org/TR/rdf-sparql-query/) Endpoint was added including a server that implements a protocol to access repositories over HTTP so that different applications can query, update and access smart device RDF observations through HTTP requests. As we will detail when describing our example scenario and its experimental implementation, in order to test a basic level of reasoning on smart device observations the SPARQL Endpoint embedded in the USN-Enabler was used also to provide support for RDF Schema inferencing.

Providing meaningful RDF representations of smart device observations and HTTP access are two of the four recommended principles in the Linked Data approach [27], namely, (1) use Uniform Resource Identifiers (URIs) to identify things; (2) use HTTP URIs so that things can be referred to and looked up (“dereferenced”); (3) provide meaningful descriptions of things using standard formats such as RDF; and (4) include links to related URIs on the Web. Linked Data constitutes a significant advance in the realization of the Semantic Web, and it has also been shown that it may become of key importance in the development of Semantic Sensor Networks [28].

To follow all four Linked Data principles the Telco USN-Platform was further extended in two complementary directions: (1) to manage smart device RDF annotations that assign URIs to different entities in the O&M-OWL model (mainly: *featuresOfInterest*, *procedure*, *observedProperty* and *location*), and (2) linking them to the large and increasing number of publicly available linked-data resources in the Semantic Web (for example, linking location URIs to dbpedia (http://dbpedia.org)). This makes it possible to publish not only observation data but also semantic links between data and formal (and often universal) descriptions based on ontologies accessible on the Semantic Web. As a result of these semantic extensions, the USN-Platform can be seen as a Semantic Sensor Network Platform.

### 4. Example Scenario: Smart Electric Car

To better illustrate the difficulties of integrating a smart device into the OGC SWE, we introduce an example of a smart electric-car, a prototype of which was evaluated experimentally. Smart cars are good representatives of future smart devices that can act both as producers and consumers of information (see, e.g., the introduction of social network services into the vehicular environment proposed in [29] as an illustration of this dual function). New generation Intelligent Transportation Systems (ITSs) should be able “both to monitor in real-time the current user location and traffic conditions, and to access the heterogeneous data sources maintained by different organizations and useful for ITS applications” [30]. Semantic technologies will be essential for the interconnection of platforms for Vehicle-to-Vehicle (V2V) and Vehicle-to-Infrastructure (V2I) real-time data exchange (see, e.g., [31]).

#### 4.1. The Scenario

In the scenario we propose, a smart electric-car device (either the connected car itself or a context-aware mobile device placed inside the car) makes use of local sensors to detect that the battery level is low. It is then able to access the Sensor Web to receive information on recent charging service times from other vehicles, real-time occupancy levels and energy prices of different nearby recharging stations, weather and road conditions, and so forth. With this information an on-board smart application could recommend the most suitable charging station. Furthermore, once the driver decides to recharge in a particular area or station, in turn this information can be published and shared through the Semantic Sensor Web, thus allowing other services to function optimally, such as smart grid management systems that would be able to keep track of, and respond to, accurate real-time information of local electricity demand (e.g., a smart grid management application could use the charge demands from all the electric cars running in a given area in order to plan the optimal distribution of the electric energy supply in view of this foreseeable demand) [32]. For this smart interaction scenario to be possible, the in-car smart device has to be able to follow the appropriate mechanisms to discover, access, and publish Sensor Web Information (implemented in the USN-Enabler).

We are considering here smart devices also as producers of human-generated observations, which is still a challenging endeavor [33]. With this in mind, looking back at Figure 4 we can think of the user of the smart device providing a “human observation” through interaction with the smart device's Human Machine Interaction (HMI) system. In our example scenario, after receiving an alert from the battery level sensor, the driver interacts with the in-car HMI system to decide to stop at a particular charging station. When the precise *StopToRecharge* information is made available in the in-car HMI, the HMI system generates the semantically annotated (*i.e.*, O&M-OWL) human generated observation. It is also the HMI system that sends the semantically annotated observation through the USN-Gateway and publishes it on the Telco USN-Platform using the USN-Enabler *InsertObservation* operation (the interested reader may see [34] for details on the particular procedure and the HMI technology we use). It is the smart device HMI system that acts as the procedure to acquire human-generated observations, and, accordingly, as can be seen in Figure 4, the *procedure* attribute in the O&M-OWL representation is the in-car HMI system, which should be described also in the SensorML model of the smart car.

#### 4.2. Performing Inferences

In this electric car scenario the SPARQL Endpoint, which is included in the semantically extended USN-Platform, can be used to store smart device RDF observations on battery-level and recharge operations in a particular geographical area. From these annotated observations a particular application, for example a smart grid application, acting as client of the USN-Platform, could derive additional high-level knowledge using inference rules. The SPARQL Endpoint included in our USN-Enabler uses a general-purpose rule engine (see Section 5), so, for example, a rule could be defined stating that if a recharge station is already supplying other cars and is close to its full capacity, *i.e.*, it is near its power limit (*Station_NearPowerLimit*), and a vehicle has enough battery charge to reach the next station (*Vehicle_AvailableCharge_for_NextStation*), then it is appropriate to advise the driver to recharge at the next station (*ReallocateVehicle*):

(1) ReallocateVehicle ← Station_NearPowerLimit & Vehicle_AvailableCharge_for_NextStation Similarly, the concepts *Station_NearPowerLimit* and *Vehicle_AvailableCharge_for_ NextStation* can be derived using other simple inference rules:
(2) Station_NearPowerLimit ← PowerDemand > PowerThreshold
(3) Vehicle_AvailableCharge_for_NextStation ← VehicleCharge > Charge_for_NextStation

Thus, let us imagine a situation in which the driver (Tom) of a specific electric car (*om:Electric_Vehicle_Tom*), receives a low battery warning from his in-car HMI system and, interacting with it, he decides to charge in a particular station (*N-IV_Km48*). Let us suppose that in this situation the HMI system automatically produces two observations: (*om:obs_1*) specifying in which station its driver wants to charge (*RechargeStation_N-IV_Km48*, as depicted in Figure 4); and (*om:obs_2*) indicating the current battery charge level (*value_x*). The abbreviated set of RDF triples in Figure 5 would represent the recharge request observation (*om:obs_1*). (Note: *om* is used as a namespace for O&M and it is placed, with a colon, before the concepts defined in the O&M schema; concepts from the vehicle ontology contain the namespace v; and *dbpedia* represents a link from a location observation to a dbpedia URI). The vehicle charge level observation (*om:obs_2*) is similar, but with the property *v:ChargeLevel* as object of the predicate *om:observedProperty*, and with the value of the *om:result* replaced by the amount of charge left in the vehicle's battery.

Once these two RDF observations have been published by the smart car HMI system through the USN-Platform, a smart grid application may check the power limit of the particular charge station and the present vehicle demand (rule 2, above). Then, if the requested station is not able to supply energy to Tom's car, the smart grid application estimates (rule 3) whether Tom's vehicle can reach the next station (*Charge_for_NextStation*) and, if it can, it adds a new RDF triple to *om:obs_1*:

om: Electric_Vehicle_Tom rdf: type v: Vehicle_AvailableCharge_for_NextStation

In this case rule 3 will infer that Tom should be redirected to another charging station (*Reallocate_Vehicle*). This information will then be transmitted to the in-car HMI system to inform Tom that he needs to continue to the next charging station.

### 5. Experimental Implementation

The USN-Platform can be implemented in different ways depending on the needs, particularities of the infrastructure, scalability requirements and so forth. Both the USN-Enabler and the USN-Gateways are logical elements and, consequently, they can be deployed in different physical embodiments.

For the purpose of testing the extended semantic capabilities of the USN-Platform, a functional experimental prototype was implemented, mainly geared towards the design of new In-Vehicle Information Systems (IVIS) under the publicly-funded Spanish research project Mobility for Advanced Transport Networks (MARTA, www.cenitmarta.org), in which several context-aware applications were implemented for connected-car scenarios. In our experimental setup the support for the in-car smart device was provided by an On-Board Unit (OBU), implementing an OSGi (www.osgi.org) framework, on which drivers can use different context-aware smart applications accessing both local sensors (car sensors) and remote sensors (the Sensor Web). The OBU also integrated several communication technologies to support V2V, V2I as well as NGNs communications [23]. These connected car capabilities enabled the exchange of information with the Sensor Web (consuming and publishing sensor information) through the USN-Platform.

**Figure 6.**
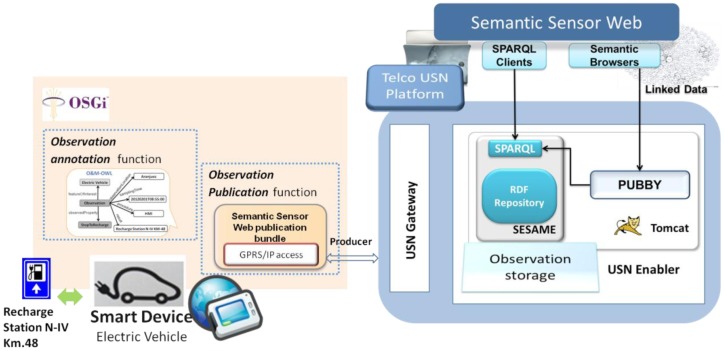
Implementation of the Telco USN-Platform to provide semantic web functionality.

As shown in Figure 6, RDF observations (O&M-OWL model) were generated by smart device applications (*i.e.*, electric vehicles) using a semantic annotation OSGi bundle, which corresponds to the *observation annotation function* described in Section 3. A “Semantic Sensor Web” publication bundle, also implemented in the smart device and corresponding to the *observation publication function* in Section 3, was then used to publish the RDF annotated observations through the IP connectivity that the USN-Gateways provide.

As discussed in Section 4, the USN-Enabler was extended to provide both storage for smart device RDF observations as well as linkage to external Semantic Web resources (see Figure 6). To achieve the first objective, the Observation Storage entity in the USN-Enabler was extended implementing the Sesame (www.openrdf.org) SPARQL Endpoint (HTTP Server and workbench Version 2.0). The HTTP server consists of a number of Java Servlets that implement a protocol to access Sesame SPARQL repositories over HTTP, while the workbench provides a web interface (Java API) for querying, updating and exploring the repositories of a Sesame Server. Thus, by implementing Sesame in the USN-Enabler, observations stored as RDF triples in said USN-Enabler can be accessed by sending SPARQL queries, and RDF resources can then be identified by means of URIs. However, these URIs used in the SPARQL repositories are not dereferenceable, meaning that, for example, they cannot be accessed from a Semantic Web browser nor, therefore, by a growing variety of Linked Data applications and clients (e.g., it is not possible to link the observation *location* to dbpedia). To tackle this limitation, and to fulfill the second objective of providing linkage to an external Semantic Web, Pubby, a Linked Data frontend for SPARQL Endpoints [35], was also implemented for the USN-Enabler. Pubby is also a web application (only requiring a servlet container such as Apache Tomcat) that provides a Linked Data interface to the RDF observations repository, mapping URIs retrieved by SPARQL endpoints to dereferenceable URIs.

Also tying into the discussion in Sections 3.2 and 4.2, a desirable feature of our USN-Enabler is to support inference on smart device observations. Sesame provides custom rule-based reasoning support and OWL inferencing by using an external inference engine, such as OWLIM (www.ontotext.com/owlim). However, for our evaluations in Section 6 only its standard RDF Schema reasoning was tested.

### 6. Evaluation of the USN-Platform Semantic Extension

Tests were conducted to evaluate the performance of the semantic extension of the USN-Platform. We analyzed the overhead of smart device observations when including semantic information, and evaluated the performance of the Observation Storage extended with the Sesame SPARQL Endpoint.

#### 6.1. Overhead of Semantic Information

First, taking as reference the original O&M language encoded in XML, we analyzed the overhead of including semantic RDF annotations into smart device observations using a RDF Scheme based on the O&M-OWL ontology and encoded also in XML. Three of the most common types of messages in our connected car use case were considered. Message A, a simple observation where an electric vehicle communicates its current battery charge level; message B, containing two observations: the specific recharge station where the vehicle wants to stop and its current charge level; and message C, containing several general vehicle observations including current velocity, acceleration, number of passengers, tire pressure, motor temperature and brake status, battery level and the desired recharge station. It is relevant to point out that all of the observations in these messages B and C are related to the same feature of interest (*i.e.*, the electric vehicle in question) and are attached to the same location and sampling time.

Table 1 compares the size (in bytes) of the three types of message with the observations represented in O&M, and also when they are semantically described using O&M-OWL (in all cases an XML format with no compression was used).

Aiming to compensate for the overhead required by O&M-OWL observations encoded in XML, we tested different compression mechanisms that are commonly used in this sort of scenario (for example when encoding SOAP XML messages exchanged by Web Services [36]). We compared some of the most common binary encoders Gzip, Fast Infoset (FI) and Efficient XML Interchange (EXI) to encode and compress the three types of message considered (A, B and C). Gzip (www.gzip.org) is a general lossless compression mechanism than can be used satisfactorily when the data has statistical redundancy. However, when the volume of information is small, Gzip does not always perform well enough with XML data, even to the point of increasing the size of the file after compression. FI [37] and EXI (www.w3.org/XML/EXI) are specifications of binary encoding of XML data, which they can represent in compact form with reasonable overhead. In addition, while Gzip is mainly directed to size optimization and the processing time for compressing and expanding files can outweigh its benefits, EXI is able to achieve not only good compression rates but also better performance than Gzip. Table 2 shows the size in bytes and the relative compression compared to the raw (*i.e.*, XML) format of each one of our three message types using Gzip, FI and EXI.

From the results in Table 1 we can see that the overhead of adding RDF annotations to O&M depends on the type of message. Message A, which contains only one observation, has a relative overhead of around 118% when including semantic information. This overhead is mainly due to the need to describe the semantic relationships between the different entities that are involved in the observation, according to the O&M-OWL ontology. However, for messages containing several observations related to the same feature of interest and linked to a same location and sampling time (as is the case of Message B and Message C), the relative overhead decreases because many RDF relationships need only to be defined once, as they are common to all of the observations. Therefore, as the values in Table 1 show, the relative overhead of their semantic description is lower than that of Message A containing a single observation.

Looking at the results in Table 2 we can conclude that despite the overhead of semantically annotated observations in O&M-OWL, by using binary encoding it is possible to reduce the size of the observations to maintain very efficient information exchanges with the USN-Platform. The best performance was achieved using the EXI encoder, which is the binary encoder that has been proposed by the W3C for smart devices (see, e.g., [38]). In consequence, we believe that the adoption of EXI in the USN-Platform will improve its performance and encourage its future use in different areas of application.

#### 6.2. Performance of the Observation Storage including a Semantic Web Repository

The experimental infrastructure used to test the performance of the semantic extension of the Observation Storage (described in Section 5), was based on a Java Servlet Container (specifically, Version 6.0 of Apache Tomcat) that supports the Sesame Java API. This container was installed in a USN-Platform back-end with an Intel Core i7 CPU 3.20 GHz processor, 12 GB of RAM and a 1.5 TB hard disk, running Windows 7 Professional x64 Service pack 1 and using Java SDK version 1.6.0_31. Additionally, we used a laptop (Intel Core i7-2640M CPU @ 2.80 GHz, 6 GB RAM, 500 GB hard disk, Windows 7 Professional x64) to simulate HTTP requests coming both from smart devices (*i.e.*, publishing observations) and USN-Platform applications (*i.e.*, semantic queries to the Observation Storage). All of the performance metrics were obtained using the capabilities that Apache JMeter (http://jmeter.apache.org/) provides, with the same Java SDK.

Using this experimental infrastructure, two complementary sets of performance tests were undertaken. First we evaluated storage performance when managing O&M-OWL observations received from smart devices. In particular, we measured the time needed to publish observations from the electric vehicle. Second, we evaluated the response times of the USN-Enabler when it provided information to applications accessing the USN-Platform; we simulated several semantic queries sent from different applications to get information related to smart car observations.

##### 6.2.1. Storage Performance

To analyze storage performance when handling semantically annotated observations sent from smart devices, two separate tests were carried out: the first consisted in measuring the time required to publish messages of varying sizes and levels of RDF inference complexity; the second was to evaluate the time required to upload sets of observations of varying sizes to the Observation Storage Sesame SPARQL Endpoint. To reiterate, only Sesame RDF Schema inferencing was tested. An important feature to consider when using Sesame is that it carries out inferences over RDF Schema when RDF triples are added to the repository (rather than performing this reasoning only when a client makes a SPARQL query). Noticeable differences in response times are thus expected when smart devices publish observations that lead to inferences of varying complexity (e.g., involving a different number of inference steps). For this reason two different configurations of Sesame (www.openrdf.org) were considered: a Native Java Store with and without RDF Schema inference capabilities.

**Figure 7.**
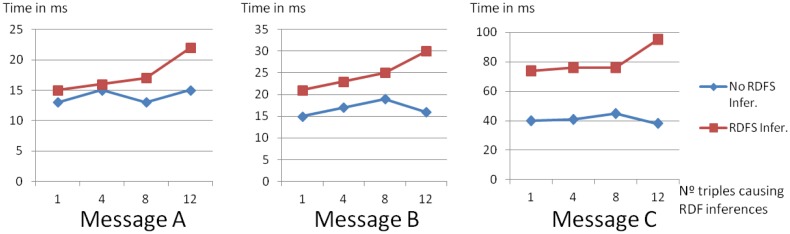
Publishing times for different types of messages, and for publication with and without RDF Schema Inference.

Figure 7 shows the publishing times for different types of message (messages A, B and C as described in 6.1) in an empty Observation Storage (to avoid any possible observation artifact caused by potential repository capacity limits). To test publishing times for different inference complexities, messages were extended with more detailed semantic descriptions of their *featureOfInterest*, requiring different amounts of RDF triples (1, 4, 8 and 12). It is clear that publishing time (*i.e.*, time-to-publication) increases with the size of the message (message C takes longer to publish than B and A for all available configurations). Figure 7 also highlights the noticeable increase in response times when using Sesame repositories with RDFS inference (red line).

**Figure 8.**
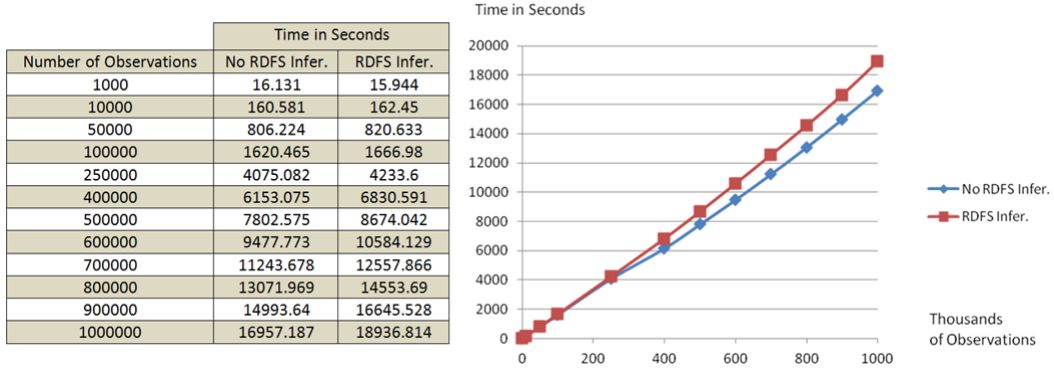
Time required to add different volumes of observations to the repositories with and without RDF Schema inference resolution.

To evaluate, now, the capacity to handle large amounts of observations, a dataset containing one million observations was created. The observations in this data set consisted in the battery levels of electric vehicles (99% of the dataset) and the power capacity of recharging stations (1% of the dataset), with an average length of 10 RDF triples per observation. Again, to evaluate Sesame's inference capabilities 20% of the observations were semantically enriched by defining a hierarchical relationship for recharging stations (*i.e.*, “v:BrandX_RechargeStation rdf:subClassOf v:RechargeStation”). The dataset was divided into subsets of different sizes and uploaded into two Sesame repositories, one with and the other without RDF inference resolution. Test results, as presented in Figure 8, show that it is possible to add 1 million smart device observations (10 million RDF triples) to our repository in 5.3 h. Moreover, the almost linear increase in uploading times reveals that, at least up to the volume of data tested, the average time needed to publish each observation (the slope in the plot in Figure 8) remains roughly constant with varying repository size.

##### 6.2.2. Accessing Information

To test the performance of the USN-Enabler when providing applications with semantic access to the USN-Platform, we measured response times to different SPARQL queries from applications processed by Sesame, again configured with and without RDFS inference resolution. We sent each query at least ten consecutive times (leaving a timeout of 1 second between each run) and computed the average response time. Three different types of SPARQL queries were used to study how response times were affected by different parameters of the repositories and queries, including *repository size*, *query complexity*, *number of returned responses*, and whether the repository accepted RDFS *inference or not*.

**Figure 9.**
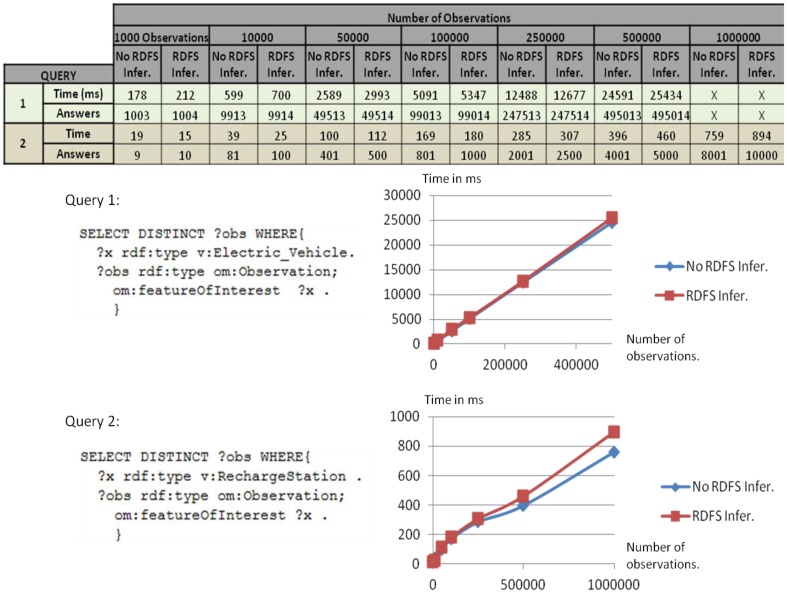
Response times for queries returning different amounts of results from the SPARQL Endpoint.

The first parameter evaluated was the response time as a function of the *number of responses to be returned*. To this end we measured response times for two queries (Queries 1 and 2) to SPARQL Endpoints of different sizes. Query 1 requested all observations whose feature of interest was an “Electric_vehicle”, and had to deal with a large number of results returned (approximately 99% of the observations stored in the repository). Query 2 searched for those observations whose feature of interest was a “Recharge_Station” (the remaining 1% of the repository), with which the number of results returned was much smaller (approximately 1%) than for Query 1. The response times obtained are presented in Figure 9 (discussion is given below).

**Figure 10.**
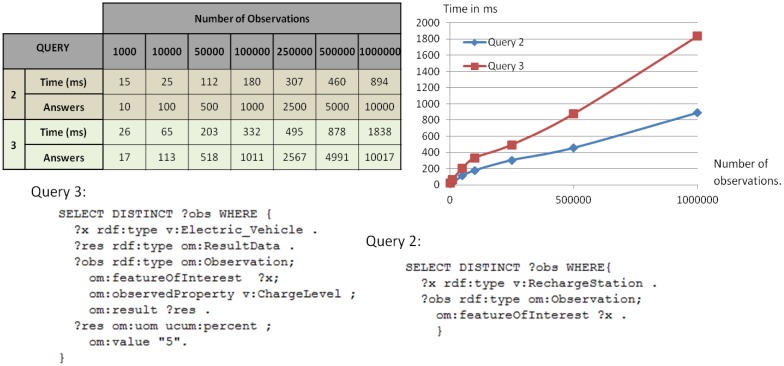
Response times for queries returning a similar number of responses but different amounts of data in the SPARQL Endpoint.

In a second test we studied the effects of *query complexity* on response times using two queries returning a similar number of results (Queries 2 and 3). Query 2 searched for all observations whose feature of interest was “RechargeStation”. Query 3 accessed “Electric_Vehicle” observations with a specific battery charge level. Figure 10 presents the response times for both queries. For any particular number of results returned we observe that response delays were significantly larger for the more complex query (Query 3), and the difference increased with the number of observations returned.

As a final test we explored how *different sizes of the repository* affect response times when the number of responses remains constant. We first stored 300 observations in the repository with the same feature of interest, observed property, location and result (value equal to 5), and then we filled it with observations similar to the previous ones but changing the observation results (result value set to 20). Once this data was available in the repository we queried it (Query 4) without RDF inference, searching for all observations with “Electric_Vehicle”, “ChargeLevel”, “Madrid” and “5” as feature of interest, observed property, location and result, respectively. Table 3 shows the results obtained for repository sizes of 1000, 10000, 50000 and 1 million observations.

Drawing from these test results we may observe, first (Figure 9), that response times increase proportionally to the number of returned responses. Moreover, the graph for Query 2 shows differences between inference and non-inference repositories. These differences are due to the higher number of returned results when using inference. In our example this is caused by the observations in the repository with “BrandX_RechargeStation” as feature of interest, which are also associated with a “RechargeStation” feature of interest through the inference from “v:BrandX_RechargeStation rdf:subClassOf v:RechargeStation”. As discussed above, inferences are made when data is loaded in the repository. Consequently, the inference process does not affect the response time, which makes most queries faster at the cost of requiring a larger repository and longer loading times. Results in Figure 10 also show how higher query complexity entails a greater response time. Finally, Table 3 shows that response times do not increase with the amount of stored data when the number of results returned is constant. This convenient result obtained using Sesame has been reported previously in other studies (see, e.g., [39]).

#### 6.3. Discussion

The results reported in this section show that publishing times are lower than 50 ms for the majority of messages, with the exception of some configurations with inference (Message C), for which the publishing times reach 100 ms. It is worth noting that these response times are well below recommended minimum separation time of 1.8 s, from leading vehicle to following vehicle, to avoid crashes caused by sudden braking [40]. The query response times obtained are less than 3% of this figure. Therefore, the publishing times in our test scenario can be considered appropriate for the delivery of emergency warning messages to motor vehicles and/or their drivers.

In this section we have also shown that the Observation Storage extended with a Sesame SPARQL endpoint can handle repository sizes of 1 million observations (around 10 million triples). This means that we can collect at least 1 million observations of electric vehicle charge levels, and make them available to a Smart Grid planning application. In addition to our evaluation results, we can also rely on other studies that have shown it is possible to add around 70 million RDF triples to a Sesame repository in 3.5 h [41]. We note that in our tests all publications and all queries were done sequentially. However, SESAME has features to facilitate scalability, such as allowing the queuing and parallel processing of incoming messages. It would be worthwhile to explore the possibility of scaling to larger amounts of data, resorting to other repository systems such as YARS2 [42], Jena TDB (http://jena.apache.org/documentation/tdb/index.html), Mulgara (http://www.mulgara.org/), Kowari (http://kowari.sourceforge.net/) or Oracle (http://www.oracle.com/technetwork/database/options/ semantic-tech/index.html).

The above results allow drawing different conclusions depending on the type of application that is requesting the information. First, some queries (Queries 2 and 3) have response times of under 100 ms when the repository size has fewer than 10,000 observations (100,000 triples). In Human Machine Interaction this magnitude of response time is considered “the limit for having the user feel that the system is reacting instantaneously, meaning that no special feedback is necessary except to display de result” [42]. Query 4 has a constant response time of around 150 ms. This response time lies within the interval of 0.1–1 s that Nielsen [43] considers “the limit for the user's flow of thought to stay uninterrupted, even though the user will notice the delay”. Query 2 has response times under 1 s for all of the repository sizes tested, and Query 3 for repositories with less than 500,000 observations (5 million triples). These delays could be acceptable in application contexts such as electric vehicles trying to locate nearby recharging stations (Query 2), or Smart Grid applications locating in an area electric vehicles with a certain level of battery charge (Queries 3 and 4). Much longer response times, of around 25 s, are obtained for Query 1 and repository sizes of 500,000 observations. These delays are inadequate for interactive applications in the context of smart devices. However, they can be perfectly acceptable for other non-real-time information systems such as applications for medium-term optimization of smart grid infrastructures (corresponding to queries which, like Query 1, wish to access all of the electric vehicles referenced in a repository).

From these experiments we can conclude that the most suitable solution for the long-term evolution of the USN-Platform is to offer both traditional and semantic functions, the latter considered as an extension of the current offering. In this manner existing applications using traditional mechanisms would continue to work without having to be altered, while future applications may decide whether semantics is appropriate for them or not (considering the increased response times for queries requiring inferences).

### 7. Conclusions

A central objective of this paper has been to discuss how Semantic Sensor Web approaches can assist in the process of integrating smart devices into future Internet-of-Things infrastructures populated by Web-accessible, heterogeneous and geographically dispersed sensor networks. We have argued that besides enjoying the benefits offered by the growing availability of Web-accessible sensor data, smart devices can also play an important role in *generating* valuable real-world information to be shared across the Semantic Sensor Web. We have taken the OGC SWE initiative as a reference of service structure to integrate smart devices into the Sensor Web, and presented a *Ubiquitous Sensor Network (USN) Platform* as a suitable framework to both integrate smart devices over Next-Generation Networks and provide efficient mechanisms to discover, access and publish real-world information. We have then explored experimentally how the USN-Platform may be extended to support Semantic Web technologies, which will allow smart devices to be more efficiently integrated with the future Semantic Sensor Web.

As a main conclusion, based on our ongoing functional and experimental activities we believe that the use of Semantic Web principles and technologies can help overcome major obstacles to integrate smart devices and real-world data. However, the scalability and efficiency of intensive distributed computing will become critical factors as the amount of data increases with the development of large-scale scenarios with connected objects (as intended for the Internet-of-Things). We will address these factors in further research.

The scalability of our Telco Platform is determined by two main features: component distribution and the current state of the art offered by big-data solutions (such as MongoDB (http://www.mongodb.org/) and Cassandra (http://cassandra.apache.org/)). These have proved to be enough for recent large-scale projects like SmartSantander. Nevertheless, the impact that the new semantic features will have on the scalability of the Platform has yet to be fully evaluated. This is the focus of part of our ongoing research efforts.

To handle the increasing number of smart devices the Telco Platform relies on NGN scalability: it requires that the devices be registered (through USN-Gateways) as user terminals. However, the impact of the specific traffic patterns generated by future IoT environments is still an open area of research. Furthermore, for highly dynamic scenarios (such as those involving “connected” cars), with the added time constraints imposed by taking into account also the interaction between the human user and the smart system, it will be necessary to develop mechanisms for information fusion, transport optimization and node mobility that can guarantee short reaction times.

The Semantic Sensor Web initiative (SSW) [44] proposes the use of ontologies and other Semantic Web technologies to enrich data from heterogeneous sensor networks. The main purpose of the SSW is to provide a unified formal representation of sensor (and sensed) data, adding semantic annotation to existing standard Sensor Web languages. There are many proposals to semantically annotate sensor observations. Among them, as reviewed by the W3C incubator group on SSN [13], XLink, which is already used in SWE documents, or RDFa, which defines attributes that can be added to SensorML and O&M documents (as we did in our previous work [45]). Acknowledging difficulties identified by the W3C SSN Incubator Group, particularly the multiple interpretations of XLink within the OGC community and the lack of a systematic way to port RDFa specifications to an XLink base, in the present paper we have followed the semantic representation of O&M that was proposed for the Semantic Sensor Observation Service (SemSOS) of Henson and colleagues [14]. In future work it will be relevant to consider the Semantic Sensor Network Ontology (SSNO), recently proposed by the W3C incubator group on SSN [13]

## Figures and Tables

**Table 1.** Sizes and relative overhead for different message types including observations with semantic information (O&M-OWL) and without it (O&M).

**Type of Message**	**O&M (bytes)**	**O&M-OWL (bytes)**	**Relative Overhead**
Message A	980	2135	117.85%
Message B	2089	3099	48.34%
Message C	6918	8747	26.43%

**Table 2.** Message sizes and percentage of compression for semantically annotated observations in O&M-OWL using Gzip, FI and EXI.

**Type of Message**	**Raw XML**	**FI**		**Gzip**		**EXI**	
	**(bytes)**	**(bytes)**	**(Compression)**	**(bytes)**	**(Compression)**	**(bytes)**	**(Compression)**
Message A	2135	1301	39.06%	683	68%	285	86.65%
Message B	3099	1621	47.69%	804	74.05%	388	87.47%
Message C	8747	3253	62.81%	1170	86.62%	903	89.67%

**Table 3.** Response times for SPARQL queries returning the same number of results from repositories of different sizes.

	**Number of Observations**			

	1000	10,000	50,000	1,000,000
**Time (ms)**	152	151	147	152
**No. Answers**	300	300	300	300
